# Complications of Intravenous Midazolam–Fentanyl Sedation in Children and Adults Undergoing Oral Surgery: A Retrospective Study

**DOI:** 10.3390/jcm14124096

**Published:** 2025-06-10

**Authors:** Margaux Nys, Melisa Garip, Ruxandra Coropciuc, Jan Meeus, Paul Legrand, Constantinus Politis

**Affiliations:** Department of Oral and Maxillofacial Surgery, University Hospitals Leuven, 3000 Leuven, Belgium

**Keywords:** intravenous sedation, adult, child, complications, hypnotics and sedatives, oral surgical procedures

## Abstract

**Objective:** This study examines the incidence and predictors of complications following intravenous (IV) sedation in children and adults. **Methods:** A retrospective analysis of 1463 surgical procedures under IV sedation was conducted at the University Hospitals of Leuven (2018–2022). Patients aged 10–91 years were divided into pediatric (10–16 years, *n* = 731) and adult (17–91 years, *n* = 732) groups. Data were analyzed using multiple regression models (*p* < 0.05). **Results:** Side effects occurred more often during recovery (children: 20.1%, adults: 9.4%) than intraoperatively (children: 4.8%, adults: 2.7%). The most common side effects were nausea (children: 10.5%, adults: 8.4%) and prolonged sedation (children: 6.0%, adults: 1.8%). Younger children had higher risks of intraoperative side effects (*p* = 0.02), hypotension (*p* < 0.001), and longer recovery (*p* < 0.001). Ketamine increased nausea risk in children (*p* = 0.02). Females had a higher risk of prolonged sedation (*p* = 0.03) and nausea (*p* = 0.01). Older adults had fewer recovery-related side effects (*p* = 0.03) and shorter recovery times (*p* = 0.05). **Conclusions:** IV sedation is a safe alternative to general anesthesia in oral surgery when properly monitored. However, nausea and prolonged sedation remain concerns, particularly in younger children and females. Prophylactic anti-emetics and cautious Ketamine use may help mitigate risks.

## 1. Introduction

Procedural sedation or conscious sedation, commonly used in dentistry and oral surgery, aims to alleviate anxiety and pain while preserving patients’ independent cardiorespiratory function and responsiveness to verbal commands [1,2,3]. While dentists frequently utilize peroral or nitrous oxide sedation, oral and maxillofacial surgeons often prefer intravenous (IV) sedation due to its predictability, safety, and rapid onset and offset of action. Efficient analgesia and reduction of anxiety result in great parental and patient satisfaction [3]. However, IV sedation is not without challenges, particularly in pediatric patients or those with needle phobia [4].

An ideal sedative should offer painless administration and rapid, predictable and efficient action, with minimal side effects [3]. Proper monitoring of capnography, pulse oximetry, respiratory rate, heart rate and blood pressure is crucial for early detection and prevention of complications [1,4]. Common side effects of sedatives include nausea [3,5,6,7], vomiting [3,5,6,7], behavioral changes [3,6,7], headaches [6], balance and gait disturbances [6], sleep disorders and nightmares [6], hallucinations [6], lethargia [6], hypotension [8], bradycardia [9] and desaturation [2,3,7,10].

This study aims to describe the incidence and predictors of complications and side effects following IV sedation in both children and adults, focusing on the impact of factors such as sex, age, the type of surgery, and the doses of Midazolam, Fentanyl and Ketamine.

## 2. Materials and Methods

### 2.1. Ethical Approval

Ethical approval was obtained from the Ethics Committee of the University Hospitals of Leuven (MP020019).

### 2.2. Population and Study Design

This retrospective, single-center cohort study included 1403 patients who underwent 1463 intraoral surgical procedures under IV sedation at the University Hospitals of Leuven between 1 January 2018, and 31 December 2022. All eligible patients within this time frame were included. No formal sample size calculation was performed. Patients were excluded if their sedation forms, including perioperative and postoperative parameters, were unavailable. The inclusion criteria were treatment at UZ Leuven, surgery under IV sedation, a minimum age of 10 years and an ASA score of 1 or 2. Patients were excluded if they had incomplete records, were under 10 years of age or had an ASA score of 3 or higher. The study followed STROBE guidelines [11].

### 2.3. Data Extraction

Data extracted from medical charts included patient gender, age, type of surgery, doses of Midazolam and Fentanyl, and the use of Ketamine. Additionally, the administration of other drugs such as methylprednisolone, ketorolac and paracetamol was recorded. The outcomes analyzed were perioperative complications and complications during recovery, including nausea, bradycardia, desaturation, hypotension, prolonged sedation and duration of recovery. All complications were identified through manual review. For each patient, the full electronic health record—including anesthesia forms, nursing documentation and postoperative reports—was reviewed. Complications were explicitly documented and manually recorded.

### 2.4. Definitions

Bradycardia is defined as a heart rate of less than 50 beats per minute [2]. Desaturation was defined as a drop in oxygen saturation exceeding 5% from the preoperative baseline [1]. Hypotension was defined as a systolic blood pressure below 80 mmHg and a diastolic pressure below 50 mmHg [8]. Prolonged sedation was measured using a sedation scale ranging from 1 to 4, evaluated every 15 min by a qualified nurse. A score of 1 indicated deep sedation without spontaneous interaction; if this score persisted after 45 min, it was classified as prolonged sedation.

Procedures were categorized into five types: third molar extractions, other tooth extractions, surgical exposure of impacted teeth, placement or removal of bone anchors or implants, and other surgeries. The “other surgeries” category included tooth autotransplantation, cystectomy, removal of a cementoma or arteriovenous malformation, sialoendoscopy, exploration, curettage and rinsing of cystic pathology, ranula enucleation, incision and drainage of an abscess, and abutment placement on a dental implant.

### 2.5. Sedation Protocol

In our department at University Hospitals Leuven, a standard protocol is followed, based on and approved by the guideline produced by the Belgian Sedation Board [12].

All patients, aged 10 years or older with an ASA score of 1 or 2, received IV Midazolam and Fentanyl under continuous monitoring of vital parameters. Additionally, patients received intravenous methylprednisolone, ketorolac and paracetamol. Dosages were consistent with departmental standards.

### 2.6. Statistical Analysis

The incidence of complications was calculated. Multiple logistic and linear regression analyses were conducted on the extracted data to identify predictors for binary and continuous outcomes, respectively. *p*-values < 0.05 were considered significant (α = 0.05). Variables included in the models were age, gender, ASA classification, and type of procedure. Covariates were selected based on clinical relevance and literature review, and those with a univariate *p*-value < 0.10 were included in the multivariate analysis. Multicollinearity was assessed using the Variance Inflation Factor (VIF); a VIF > 5 was considered indicative of problematic collinearity and handled by excluding or combining variables. Analyses were performed using IBM SPSS Statistics version 28.0.0.0 (IBM corp, Armonk, NY, USA).

The duration of surgery was not available in the electronic records and could not be included in the analyses. This limitation is acknowledged and discussed below.

## 3. Results

### 3.1. Patient and Procedure Characteristics

Patients were divided into two age groups: pediatric (10–16 years) and adult (17–91 years). The pediatric group included 335 procedures in 286 boys (45.8%) and 396 procedures in 336 girls (54.2%), with an average age of 14.34 ± 1.64 years. The adult group included 302 procedures in 301 men (41.3%) and 430 procedures in 416 women (58.7%), with an average age of 27.5 ± 19.8 years. Patient and procedure characteristics are shown in Table 1.

### 3.2. Incidence of Complications and Side Effects

Table 2 provides an overview of the incidences of the different complications and side effects per group. A complication is described in the literature as an unintended and undesired event or condition that occurs during or following a medical intervention and causes temporary or irreversible damage to the patient’s health. Since none of the events could be defined as a complication, we renamed them as side effects. All events could be attributed to known possible side effects of the administered medication.

#### 3.2.1. Children

In the pediatric group, 24.2% of procedures resulted in side effects, occurring more frequently during recovery (20.1%) than intraoperatively (4.8%). The most common side effects were nausea (10.5%), prolonged sedation (6.0%) and hypotension (4.5%). Desaturation (3.1%) and bradycardia (1.9%) were less common. The average recovery time was 60.4 ± 30.6 min.

#### 3.2.2. Adults

In the adult group, 11.6% of procedures resulted in side effects, with more occurrences during recovery (9.4%) than intraoperatively (2.7%). The most common side effects were nausea (8.4%) and prolonged sedation (1.8%), while desaturation (0.5%) and hypotension (0.4%) were rare. The average recovery time was 35.2 ± 17.4 min.

### 3.3. Multiple Logistic Regression Analysis

The results of the multiple logistic regression analysis can be found in Table 3.

#### 3.3.1. Children

Younger age (OR = 0.74; *p* = 0.02) and a higher dose of Fentanyl (OR = 1.15; *p* = 0.05) were significant predictors for intraoperative side effects. Younger age was also significantly associated with hypotension (OR = 0.60; *p* < 0.001). The administration of Ketamine significantly increased the risk of nausea (OR = 31.88; *p* = 0.02). In conclusion, girls have an increased risk for prolonged sedation (OR = 2.12; *p* = 0.03) and a decreased risk for desaturation (OR = 0.36; *p* = 0.03) compared to boys.

#### 3.3.2. Adults

In adults, increasing age was associated with a reduced risk of complications during recovery (OR = 0.96; *p* = 0.03), while female sex was associated with a higher risk of recovery-related complications (OR = 2.08; *p* = 0.01), particularly nausea (OR = 1.91; *p* = 0.02). Finally, higher age tends to be protective against nausea (OR = 0.95; *p* = 0.03).

### 3.4. Multiple Linear Regression Analysis

The results of the multiple linear regression analysis can be found in Table 4.

In children, increasing age was significantly associated with a shorter recovery duration (*p* < 0.001). In adults, no significant correlation was found between age and recovery duration (*p* = 0.05).

## 4. Discussion

This study highlights seven key findings. First, side effects occurred more frequently during recovery than during the surgical procedure. Second, nausea and prolonged sedation were the most common side effects in both pediatric and adult groups. In children, these were followed by hypotension, desaturation and bradycardia, while in adults, desaturation and hypotension were rare. Third, younger age and higher doses of Fentanyl were significant predictors for intraoperative side effects in children. Fourth, younger age was associated with an increased risk of hypotension and a prolonged stay in recovery. Fifth, Ketamine administration in children significantly increased the risk of nausea, although this finding should be interpreted cautiously due to the small number of patients receiving Ketamine. Sixth, females were at higher risk for nausea, likely due to the emetogenic effects of progesterone. This explains the difference between men and women and between pre- and postmenstrual age [13]. Some studies confirmed the influence of female reproductive hormones on the gastrointestinal tract, where progesterone inhibits gastric motility and therefore causes nausea and emesis [14]. Lastly, no patient was given an antidote for the administered sedatives, indicating the safety of this protocol if implemented correctly. Nausea and prolonged sedation remain among the most frequently reported adverse events, with rates similar to previous findings by Alletag et al. [3] and McCarty et al. [7]. The influence of age and gender on these outcomes is consistent with pharmacodynamic expectations and hormone-related mechanisms as reported by Zou et al. [13].

In the pediatric population, a significant association between younger age and hypotension was demonstrated. This can be explained by the physiologic age-related difference in blood pressure in the pediatric population, which has not been considered in defining hypotension in this study [15].

Among children, an increased incidence of nausea was demonstrated with the use of Ketamine in addition to Midazolam and Fentanyl. In our study, the emetogenic effect of Ketamine (OR = 31.882) might have been falsely enhanced because only six patients received Ketamine. As a result, this outcome should be interpreted with care. However, a clear trend has been demonstrated, and nausea is a well-described side effect of Ketamine in the literature [3,5,7]. The incidence of nausea after the administration of Ketamine for sedation in a pediatric population is 7–26% [3]. The emetogenic effect of Ketamine is not dose-related [3]. The administration of Atropine in addition to Ketamine has a small anti-emetic effect with a number needed to treat of 16 [5] This effect is comparable to the prophylactic administration of Ondansetron [16].

In the adult population, bradycardia, desaturation and hypotension occurred only in 1.0%, 0.5% and 0.4% of patients, respectively. Statistical analyses would thus be underpowered, and as a result, no analyses were conducted to uncover possible risk factors for these side effects. Since only one patient in the adult group received Ketamine, this could not be included as a possible predictor in the analyses for the abovementioned reason.

As mentioned before, no major complications occurred. Therapeutic intervention consisted of admission of atropine, anti-emetic medication or oxygen with a maximum of 4 L per minute. Therefore, we like to define these events as side effects rather than complications, since these are medication-related events and could be easily treated.

This study’s strengths include the large patient population, the use of multivariate analysis to control for confounding factors, and the consistency of the sedation protocol.

However, we also acknowledge several limitations. First, all patients with an American Society of Anesthesiology (ASA) score higher than 2 were referred to another campus of the University Hospitals of Leuven for surgery in the presence of an anesthesiologist, and therefore, they were not enrolled in this study. This contributes to a certain degree of selection bias. Second, the follow-up period of this study is limited to time to discharge. As a result, we cannot draw conclusions on the risk for complications after discharge, and the incidences might be underestimated. However, we would expect the included complications to occur early. Third, as mentioned before, the association between the use of Ketamine and the incidence of nausea in the pediatric population should be interpreted with care, since only six patients received Ketamine in addition to Midazolam and Fentanyl. Fourth, surgical duration was not consistently recorded and could not be analyzed as a potential confounder. Lastly, the study depends on the accuracy and consistency of clinician documentation in medical records, which may vary.

## 5. Conclusions

Anxiolysis by IV sedation for oral surgery is a safe alternative to general anesthesia in children and adults when appropriately indicated.

However, consideration should be given to the risk of nausea and prolonged sedation after intravenous sedation in a pediatric and adult population. The prophylactic administration of anti-emetic medication may be considered a useful measure in the prevention of nausea after intravenous sedation, especially in women since they are more prone to nausea due to progesterone levels.

## Figures and Tables

**Table 1:** Patient and procedure characteristics.

A: Children	
Gender	Boys: 45.8% (*n* = 335) Girls: 54.2% (*n* = 396) Missing: 0.0% (*n* = 0)
Type of surgery	Third molar extractions: 62.7% (*n* = 458) Tooth extractions (other than third molars): 20.2% (*n* = 148) Impacted tooth exposure: 6.2% (*n* = 45) Bone anchor placement: 7.4% (*n* = 54) Other surgery: 3.3% (*n* = 24) Missing: 0.3% (*n* = 2)
Use of Ketamine	No: 99.2% (725) Yes: 0.8% (*n* = 6) Missing: 0.0% (*n* = 0)
**B: Adults**	
Gender	Male: 41.3% (*n* = 302) Female: 58.7% (*n* = 430) Missing: 0.0% (*n* = 0)
Type of surgery	Third molar extractions: 84.2% (*n* = 616) Tooth extractions (other than third molars): 5.2% (*n* = 38) Implants: 1.9% (*n* = 14) Other surgery: 8.7% (*n* = 64) Missing: 0.0% (*n* = 0)
Use of Ketamine	No: 99.9% (*n* = 731) Yes: 0.1% (*n* = 1) Missing: 0.0% (*n* = 0)

**Table 2:** Incidence of side effects.

A: Children	
Complication	Incidence
Perioperative complications	4.8% (*n* = 35)
Complications during recovery	20.1% (*n* = 147)
Nausea	10.5% (*n* = 77)
Bradycardia	1.9% (*n* = 14)
Desaturation	3.1% (*n* = 23)
Hypotension	4.5% (*n* = 33)
Prolonged sedation	6.0% (*n* = 44)
**B: Adults**	
Complication	Incidence
Perioperative complications	2.7% (*n* = 20)
Complications during recovery	9.4% (*n* = 69)
Nausea	8.9% (*n* = 65)
Bradycardia	1.0% (*n* = 7)
Desaturation	0.5% (*n* = 4)
Hypotension	0.4% (*n* = 3)
Prolonged sedation	1.9% (*n* = 14)

**Table 3:** Multiple logistic regression analysis.

A: Children		
Perioperative events		
Risk Factors	*p*-Value	Odds Ratio
Gender	0.064	
Age	**0.020**	0.735
Type of surgery	0.607	
Dose of Midazolam	0.715	
Dose of Fentanyl	**0.046**	1.147
Use of Ketamine	0.999	
Events during recovery		
Risk Factors	*p*-Value	Odds Ratio
Gender	0.156	
Age	0.231	
Type of surgery	0.095	
Dose of Midazolam	0.736	
Dose of Fentanyl	0.660	
Use of Ketamine	0.098	
Nausea		
Risk Factors	*p*-Value	Odds Ratio
Gender	0.062	
Age	0.682	
Type of surgery	0.411	
Dose of Midazolam	0.194	
Dose of Fentanyl	0.087	
Use of Ketamine	**0.020**	31.882
Bradycardia		
Risk Factors	*p*-Value	Odds Ratio
Gender	0.971	
Age	0.948	
Type of surgery	0.752	
Dose of Midazolam	0.874	
Dose of Fentanyl	0.507	
Use of Ketamine	0.999	
Desaturation		
Risk Factors	*p*-Value	Odds Ratio
Gender	**0.031**	0.364
Age	0.653	
Type of surgery	0.714	
Dose of Midazolam	0.646	
Dose of Fentanyl	0.857	
Use of Ketamine	0.999	
Hypotension		
Risk Factors	*p*-Value	Odds Ratio
Gender	0.413	
Age	**<0.001**	0.600
Type of surgery	0.968	
Dose of Midazolam	0.267	
Dose of Fentanyl	0.446	
Use of Ketamine	0.999	
Prolonged sedation		
Risk Factors	*p*-Value	Odds Ratio
Gender	**0.031**	2.119
Age	0.129	
Type of surgery	0.082	
Dose of Midazolam	0.603	
Dose of Fentanyl	0.182	
Use of Ketamine	0.083	
**B: Adults**		
Perioperative events		
Risk Factors	*p*-Value	Odds Ratio
Gender	0.423	
Age	0.237	
ASA score	0.865	
Type of surgery	0.569	
Dose of Midazolam	0.163	
Dose of Fentanyl	0.552	
Events during recovery		
Risk Factors	*p*-Value	Odds Ratio
Gender	**0.010**	**2.078**
Age	**0.029**	**0.963**
ASA score	0.911	
Type of surgery	0.557	
Dose of Midazolam	0.870	
Dose of Fentanyl	0.148	
Nausea		
Risk Factors	*p*-Value	Odds Ratio
Gender	**0.024**	**1.910**
Age	**0.026**	**0.953**
ASA score	0.961	
Type of surgery	0.563	
Dose of Midazolam	0.772	
Dose of Fentanyl	0.286	
Prolonged sedation		
Risk Factors	*p*-Value	Odds Ratio
Gender	0.134	
Age	0.409	
ASA score	0.608	
Type of surgery	**0.020**	
Dose of Midazolam	0.349	
Dose of Fentanyl	0.779	

Bold numbers represent a statistically significant number.

**Table 4:** Multiple linear regression analysis.

A: Children		
Time in recovery		
Risk Factors	*p*-Value	Odds Ratio
Gender	0.198	
Age	**<0.001**	
Type of surgery	0.765	
Dose of Midazolam	0.750	
Dose of Fentanyl	0.856	
Use of Ketamine	0.262	
**B: Adults**		
Time in recovery		
Risk Factors		*p*-Value
Gender		0.826
Age		**0.051**
ASA score 2 (compared to 1)		0.052
ASA score 3 (compared to 1)		0.859
Type of surgery: extractions other than third molars (compared to third molar extractions)		**0.045**
Type of surgery: implants (compared to third molar extractions)		0.147
Type of surgery: other (compared to third molar extractions)		**0.002**
Dose of Midazolam		0.722
Dose of Fentanyl		0.977

Bold numbers are statistically significant numbers.

## Data Availability

The data presented in this study are available on request from the corresponding author due to ethical and privacy reasons. Data are stored on a secure server that is the property of the University Hospitals of Leuven.
